# Multiparametric MRI radiomics fusion for predicting the response and shrinkage pattern to neoadjuvant chemotherapy in breast cancer

**DOI:** 10.3389/fonc.2023.1057841

**Published:** 2023-05-03

**Authors:** Ming Fan, Xilin Wu, Jiadong Yu, Yueyue Liu, Kailang Wang, Tailong Xue, Tieyong Zeng, Shujun Chen, Lihua Li

**Affiliations:** ^1^Institute of Biomedical Engineering and Instrumentation, Hangzhou Dianzi University, Hangzhou, China; ^2^Department of Mathematics, The Chinese University of Hong Kong, Shatin, Hong Kong SAR, China; ^3^Zhejiang Cancer Hospital, Institute of Basic Medicine and Cancer (IBMC), Chinese Academy of Sciences, Hangzhou, Zhejiang, China

**Keywords:** breast cancer, radiomics, multiparametric MRI, neoadjuvant chemotherapy, concentric shrinkage

## Abstract

**Purpose:**

During neoadjuvant chemotherapy (NACT), breast tumor morphological and vascular characteristics are usually changed. This study aimed to evaluate the tumor shrinkage pattern and response to NACT by preoperative multiparametric magnetic resonance imaging (MRI), including dynamic contrast-enhanced MRI (DCE-MRI), diffuse weighted imaging (DWI) and T2 weighted imaging (T2WI).

**Method:**

In this retrospective analysis, female patients with unilateral unifocal primary breast cancer were included for predicting tumor pathologic/clinical response to NACT (n=216, development set, n=151 and validation set, n=65) and for discriminating the tumor concentric shrinkage (CS) pattern from the others (n=193; development set, n=135 and validation set, n=58). Radiomic features (n=102) of first-order statistical, morphological and textural features were calculated on tumors from the multiparametric MRI. Single- and multiparametric image-based features were assessed separately and were further combined to feed into a random forest-based predictive model. The predictive model was trained in the testing set and assessed on the testing dataset with an area under the curve (AUC). Molecular subtype information and radiomic features were fused to enhance the predictive performance.

**Results:**

The DCE-MRI-based model showed higher performance (AUCs of 0.919, 0.830 and 0.825 for tumor pathologic response, clinical response and tumor shrinkage patterns, respectively) than either the T2WI or the ADC image-based model. An increased prediction performance was achieved by a model with multiparametric MRI radiomic feature fusion.

**Conclusions:**

All these results demonstrated that multiparametric MRI features and their information fusion could be of important clinical value for the preoperative prediction of treatment response and shrinkage pattern.

## Introduction

Neoadjuvant chemotherapy (NACT) is routinely used to treat locally advanced tumors to be operable, allowing for breast-preserving surgery (1, 2). Patients who achieve pathologic complete response (pCR) to NACT tend to have improved disease-free and overall survival compared with patients with residual invasive disease (3–5). Nevertheless, only a subset (varying from 10% to 25%) of the patients achieved pCR after NACT, as reported by a larger meta-analysis with 3776 patients (6). Therefore, predicting the response to NACT before treatment is important for the accurate management of breast cancer.

During NACT, breast cancers can also show different regression/shrinkage patterns that are associated with breast cancer treatment outcomes (7). Tumor concentric shrinkage (CS) is associated with good survival in low-grade breast cancer (8) and is regarded as a more suitable marker than pCR to choose candidates for breast-conserving surgery (1). Therefore, the preoperative identification of patients who have a sufficient response to NACT and how the tumor changes during treatment are indispensable in guiding chemotherapy and surgical treatment (9).

Existing studies have attempted to predict the tumor response to NACT before treatment using a magnetic resonance imaging (MRI)-based predictive model (10–12). Radiomics extracted from DCE-MRI was used to predict tumor response in breast cancer (13–15). In addition to DCE-MRI, T2-weighted imaging (T2WI) and apparent diffusion coefficient (ADC) derived from diffuse weighted imaging (DWI) are also used as predictors for evaluating NACT responses (16, 17).

To increase the prediction accuracy, multiparametric MRI (DCE-MRI, T2WI and ADC) features were analyzed and/or combined (18–20). Specifically, DCE-MRI and T2WI radiomics were implemented, which showed that tumor morphologic and quantitative enhancement kinetics can increase the specificity in predicting breast cancer treatment responses (21). Texture analysis of tumors from T2WI and ADC has been conducted for predicting recurrence in breast cancer patients treated with NACT and surgery (22). The addition of histological information of hormone receptor status, Ki-67 index, and MRI variables with radiomics were also used, which showed enhanced discrimination power in predicting tumor response (23).

Despite these advances, how multiparametric images correlate with the tumor response to NACT and the shrinkage pattern still need to be explored. In this study, we sought to enhance the prediction of tumor shrinkage patterns and responses after full cycles of treatment by using radiomics from multiparametric MR image maps and to comprehensively investigate the effectiveness of various parametric images in prediction.

## Materials and methods

### Patients

**Figure 1:**
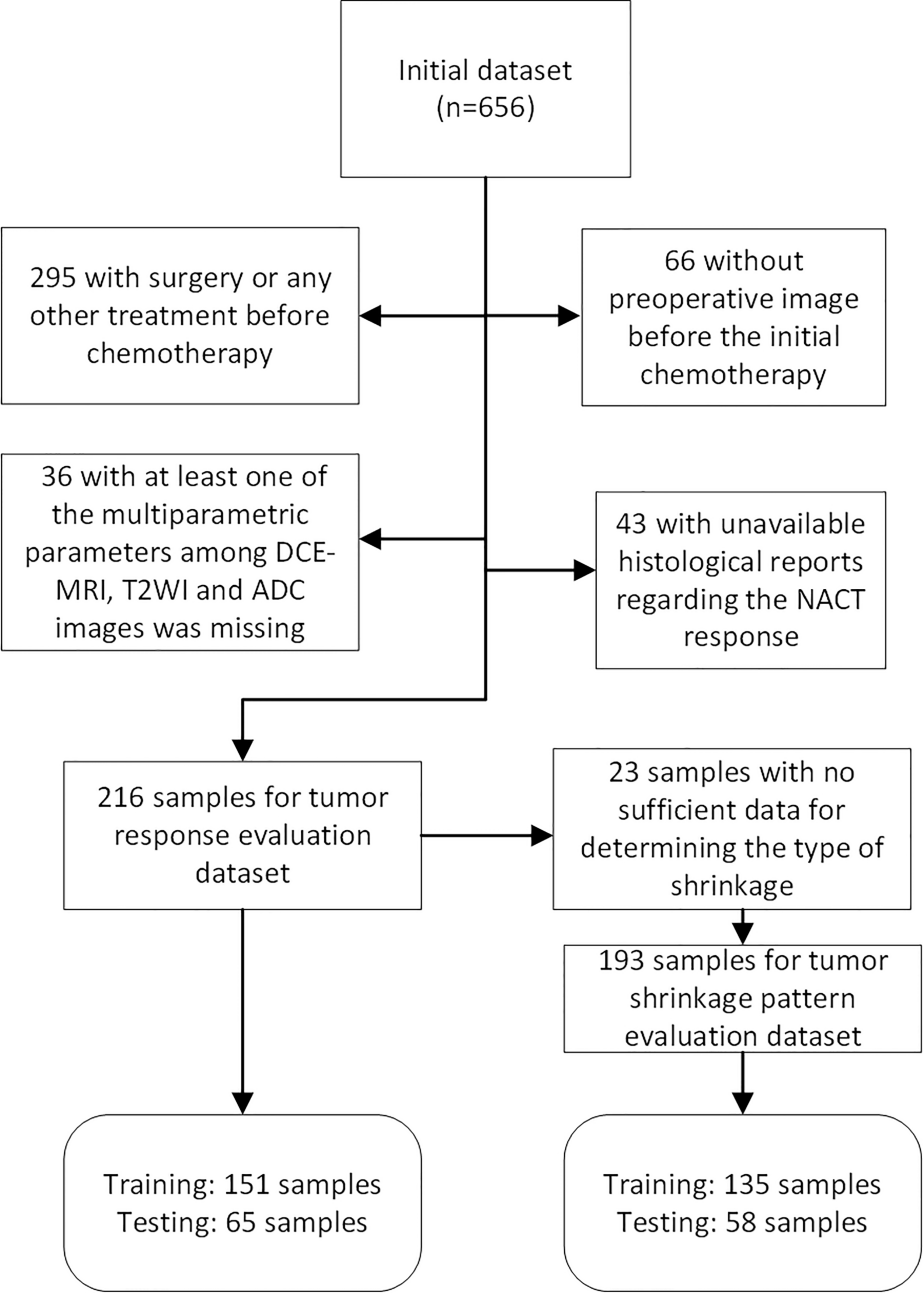
Flow chart of patients included and excluded in tumor pathologic/clinical response and shrinkage pattern datasets.

This retrospective study, which was approved by the Institutional Review Board, initially included 656 female unilateral breast cancer patients. A total of 216 and 193 unilateral unifocal primary breast cancer patients remained for evaluation of response to NACT and shrinkage pattern (**Figure 1**) after the selection with an exclusion criterion as follows: 1) surgery or any other treatment before chemotherapy (n=295); 2) at least one of the images among DCE-MRI, T2WI and DWI was missing (n=36); 3) no preoperative image before the initial chemotherapy (n=66); and 4) histopathologic information for residual tumor size was not available (n=43). Additionally, 23 samples were further excluded from the tumor shrinkage pattern analysis dataset because the follow-up data during treatment were not available for determining the type of shrinkage.

### Treatment response evaluation

All patients underwent standard NACT treatment with six to eight cycles of taxotere–epirubicin–cyclophosphamide (TEC) regimen (24). The duration of the NACT is about 4 or 5 months, and the time interval between the treatments is approximately 20 days. The pCR to NACT was defined as the absence of residual cancer in the surgical specimen. After NACT, 25 patients achieved pCR and 191 patients were non-pCR.

Tumor clinical response was evaluated by monitoring the changes in the longest diameter (LD) of the tumor and kinetic features proposed in the Response Evaluation Criteria in Solid Tumors (RECIST) 1.1 guidelines, which are widely used as recommended tumor measurements (25). According to these guidelines, patients were categorized as having a complete response (CR), partial response (PR), stable disease (SD), or progressive disease (PD). A PR was defined as at least a 30% decrease in the longest tumor diameter (LD). PD indicated an increase of less than 20% or a decrease of less than 30% in the LD. After the completion of the whole treatment procedure, 34 patients achieved CR, 156 achieved PR, one achieved PD and 25 achieved SD. Similar to a previous study, clinical responders (n=190) were defined as PR and CR, and non-clinical responders were defined as PD and SR (n=26) (13).

For tumor shrinkage evaluation, the pattern of shrinkage by NACT can be categorized as CS and non-CS (8). The CS pattern was defined as 1) only reduced tumor size and no small mass surrounding the main tumor or only small foci (less than 5 mm) surrounding the main tumor (26) and 2) CR after treatment. The non-CS pattern was defined as a tumor with 1) diffuse decrease; 2) decrease in intensity only; and 3) no change or enlargement (i.e., PD or SD). Patients in the tumor shrinkage dataset (n=193) were grouped as CS (n=166) and non-CS (n=27).

### MRI acquisition

All patients received preoperative MRI examinations in a prone position using a 3.0 T scan system (Siemens Medical Systems, Erlangen, Germany) with a dedicated eight-channel breast array coil. Fat-suppressed bilateral sagittal three-dimensional T2WI, DWI and DCE-MRI were acquired for each patient. For DCE-MRI, one precontract image (S0) followed by five postcontrast images (from S1 to S5) was obtained after injection of contrast agent with a bolus of 0.1 mmol/kg gadobutrol at a rate of 2 mL/second. The time interval between the precontrast and postcontrast image series was 60 seconds. ADC maps were generated from DWIs at b values of 50 and 1000 s/mm^2^. Detailed MR imaging parameters for DCE-MRI, DWI and T2WI are illustrated in **Supplementary Table 1**.

### Image preprocessing

Image normalization was performed and the pixel value was mapped to a fixed gray area ranging from 0 to 800. A fuzzy C-mean clustering method was used to segment the tumor region of interest (ROI) on DCE-MRI at the intermediate postcontrast series that usually has the highest enhancement to facilitate visual inspection (27). To minimize segmentation errors, the obtained tumor ROI was examined and manually corrected by our investigator. Thereafter, the generated tumor ROI on the DCE-MRI was aligned to the DWI with a b value of 50 and the T2WI map using the Statistical Parametric Modeling toolbox version 12 (SPM12) platform (http://www.fil.ion.ucl.ac.uk/spm/software/spm12). The aligned tumor ROI in DWI was mapped to the ADC image. The precontrast (S0) and first to fifth postcontrast images (termed S1 to S5) were obtained. The subtraction maps, namely, the subtraction between the first, second, third and fifth postcontrast images and S0 (termed S10, S20, S30 and S50, respectively) and that generated by subtraction between the fifth and second postcontrast images (termed S52), were analyzed.

### Radiomic feature analysis

Radiomic features (n=102) were extracted from each image series using the publicly available software package pyradiomics (28). These features include morphologic (n=14), first-order statistics (n=18), and texture features (n=70). Specifically, texture features include the gray level cooccurrence matrix (GLCM) (n=24), the gray-level run-length matrix (GLRLM) (n=16), the gray-level size-zone matrix (GLSZM) (n=16) and the gray-level spatial dependence matrix (GLDM) (n=14)-based features. A detailed list of the features is illustrated in **Supplementary Table 2**. Tumor radiomics was extracted on the DCE-MRI series (from S1 to S5), the subtraction maps (S10, S20, S30, S50 and S52) and the ADC and T2W images.

### Pathological examination

The status of the estrogen receptor (ER), progesterone receptor (PR), human epidermal growth factor receptor (HER2), and Ki-67 expression level was acquired by using streptavidin-peroxidase (SP) immunohistochemistry (29). Hormone receptor (HR) positivity was determined as ER- or PR-positive, and negativity was defined as both ER- and PR-negative. Ki-67 expression levels greater than 14% were determined to be positive. Breast cancer subtypes were determined as follows: luminal A, HR positive and HER2 negative and low Ki-67 expression level; luminal B, HR positive and either HER2 positive or HER2 negative with high levels of Ki-67; basal like, HR negative and HER2 negative; and HER2-enriched, HR negative and HER2 positive.

### Statistical analysis

The differences in the categorized variables, i.e., the histological characteristics between the groups (pCR and non-pCR; CS and non-CS groups; clinical response and non-clinical response), were evaluated using a $\chi^{2}$ test. The difference between the continuous values, i.e., age, was evaluated using analysis of variance (ANOVA). Each molecular subtype information was binarized, which was one if it was a particular subtype and zero if it was any other subtype.

A recursive feature elimination (RFE) method was used to rank the features that were most relevant to the target with a random forest model used as a base classifier. The ranked features were then sequentially added into the predictive model, in which the parameters were tuned using a grid search method with a 10-fold cross-validation (CV) framework. The importance of the image features in the predictive model was assessed by using the mean decrease in the Gini score of the random forest over all the CV loops.

Thereafter, the tuned model parameters with the optimal feature subset were used to establish a predictive model using all the samples in the training set. The model performance was assessed on the testing set with an area under the receiver operating characteristic (ROC) curve (AUC).

We have also provided the model performance evaluation by using a nested five-fold CV. Specifically, the inner loop was used to optimize model parameters of the random forest using grid search under ten-fold CV on the training data, while the outer loop produced a prediction score for the testing data. The DeLong test was used to compare the AUCs. The sensitivity and specificity were calculated for the ROC curve by using the Youden index to maximize their summation. P values less than 0.05 were considered significant. Statistical analysis and machine learning methods were performed using R (version 4.0) and MATLAB (MathWorks, Natick, Massachusetts, version 2018 b).

## Results

### Patient characteristics

Patient characteristics regarding the pathologic response, clinical response and tumor shrinkage pattern are illustrated in **Table 1** and **Supplementary Tables 3** and **4**, respectively. For the pathologic or clinical response dataset (n=216), samples were separated into a development (n=151) and a validation (n=65) set. The tumor shrinkage pattern dataset (n=193) was also separated into development (n=135) and validation (n=58) datasets.

**Table 1:** Patient characteristics in the tumor pathological response prediction dataset.

Characteristics	All (n=216)	Development Set (n=151)			Validation Set (n=65)		
		pCR (n=17)	Non-pCR (n=134)	P Value^*^	pCR (n=8)	Non-pCR (n=57)	P Value^*^
Age (y)				0.907^a^			0.522^a^
Range	26-67	27-65	26-65		37-60	34-67	
Median	49	48	49		49	51	
Mean ± std	49.3 ± 8.7	48.7 ± 8.5	48.9 ± 8.3		48.3 ± 7.2	50.2 ± 9.8	
Family history				0.239^c^			0.655^c^
No	167 (77%)	11	104		6	46	
Yes	49 (23%)	6	30		2	11	
Menopausal status				0.114^b^			1.000^c^
Pre	114 (53%)	13	71		4	31	
Post	102 (47%)	4	63		4	26	
Maximum tumor diameter (cm)				0.313^a^			0.594^a^
Range	0.5-14.5	1.6-7.0	1.8-14.5		2.2-7.5	0.5-8.3	
Median	3.7	3.5	3.7		4.1	3.4	
Mean ± std	4.1 ± 1.8	3.8 ± 1.5	4.2 ± 1.9		4.1 ± 1.9	3.7 ± 1.5	
Progesterone receptor				0.370^b^			0.958^c^
Positive	111 (51%)	7	75		3	26	
Negative	105 (49%)	10	59		5	31	
Estrogen receptor				0.524^b^			1.000^c^
Positive	133 (62%)	9	86		5	33	
Negative	83 (38%)	8	48		3	24	
Human epidermal growth factor receptor 2				0.117^c^			1.000^c^
Positive	83 (38%)	10	45		4	24	
Negative	117 (55%)	6	78		4	29	
Unknown	16 (7%)	1	11		0	4	
Ki-67				0.738^c^			1.000^c^
High	184 (85%)	15	109		8	52	
Low	32 (15%)	2	25		0	5	
Molecular subtypes				0.200^c^			1.000^c^
Luminal A	20 (9%)	0	18		0	2	
Luminal B	119 (55%)	9	73		5	32	
Basal-like	41 (19%)	3	25		2	11	
HER2	36 (17%)	5	18		1	12	
Lymph node				0.002^c^			0.372^c^
Positive	168 (78%)	8	110		5	45	
Negative	48 (22%)	9	24		3	12	

^*^P value for clinical response versus non-clinical response comparison. ^a^The data were tested using a t test; ^b^The data were tested using the chi-squared test. ^c^The data were tested using Fisher’s exact test.

From **Table 1** and **Supplementary Table 3**, there was no significant association between tumor NACT responses (pathologic and clinical responses) and the patient histological characteristics, including age, family history, menopausal status, maximum tumor diameter, PR status, ER status, HER2 status, Ki-67 status, molecular subtypes, and lymph node status. Moreover, no significant differences in these features were observed between the CS and non-CS groups. A significant association between the CS pattern and the tumor response was observed with a *p* value of 0.02.

### Individual parametric MRI radiomic analysis

**Figure 2:**
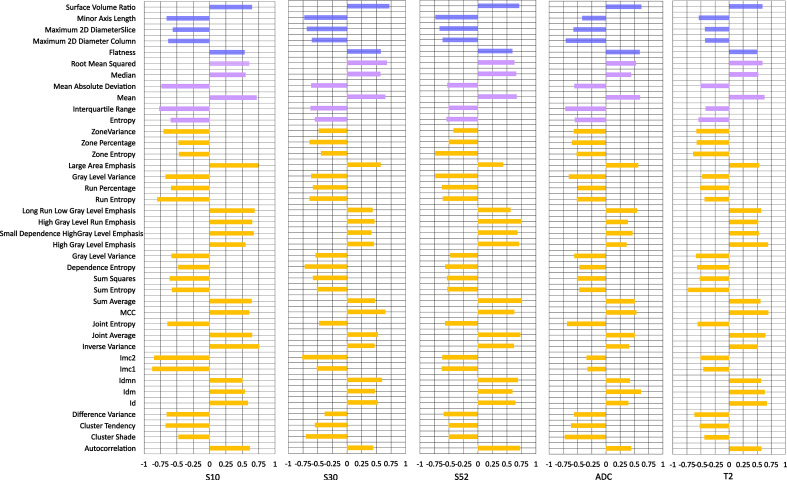
Individual feature performance for predicting pathologic response to neoadjuvant chemotherapy. The bar plot is drawn on the right side if a higher value was observed in pCR than in non-pCR, while the bar plot is drawn on the right side if the opposite was true. S10, S20, S30 and S50 represent the subtraction map between the first, second, third and fifth postcontrast images and precontrast image. S52 represents subtraction between the fifth and second postcontrast images.

Individual features associated with the tumor response and shrinkage pattern to NACT were assessed for DCE-MRI series/subtraction images and T2WI and ADC images. The higher voxel volume and the maximum 2D diameter were associated with the non-pCR and non-CS patterns of the tumor during treatment (**Figure 2**; **Supplementary Figure 1**, respectively). For DCE-MR image series or maps, individual radiomic features obtained from the subtraction images, particularly the S50 and S30 maps, are better than those obtained using the precontrast (S0) image. For parametric images other than DCE-MRI, radiomics from T2WI is relatively better than that from ADC images.

**Figure 3:**
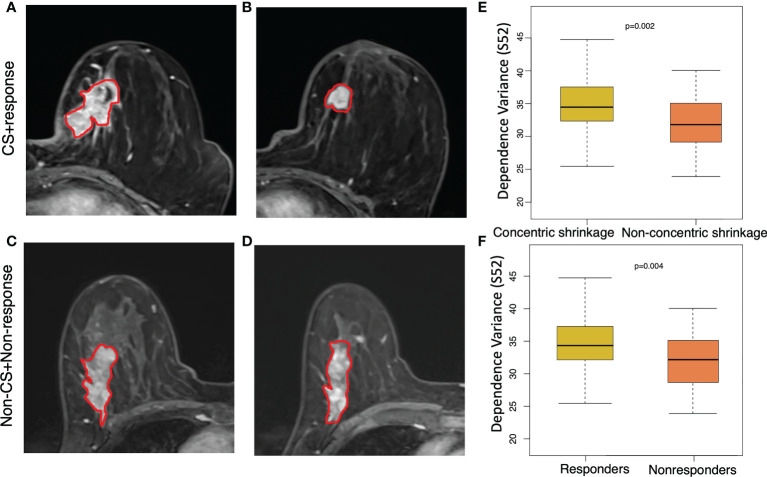
Texture feature of dependence values for discriminating concentric shrinkage (CS) pattern/response and non-CS shrinkage pattern/nonresponse tumors. This texture feature was calculated from the image subtraction between the fifth and the second postcontrast image series (S52). A 64-year-old woman with cancer in the left breast at **(A)** pretreatment and **(B)** posttreatment S52 image who responded well to chemotherapy with a CS pattern. A 65-year-old woman with cancer in the right breast at **(C)** pretreatment and **(D)** posttreatment S52 who had a poor response to chemotherapy with a non-CS pattern. The boxplot shows a significantly higher dependence variance feature value in the **(E)** clinical responder group than in the non-clinical responder group, and the same higher value was observed **(F)** in patients who were CS than in non-CS.

**Figure 4:**
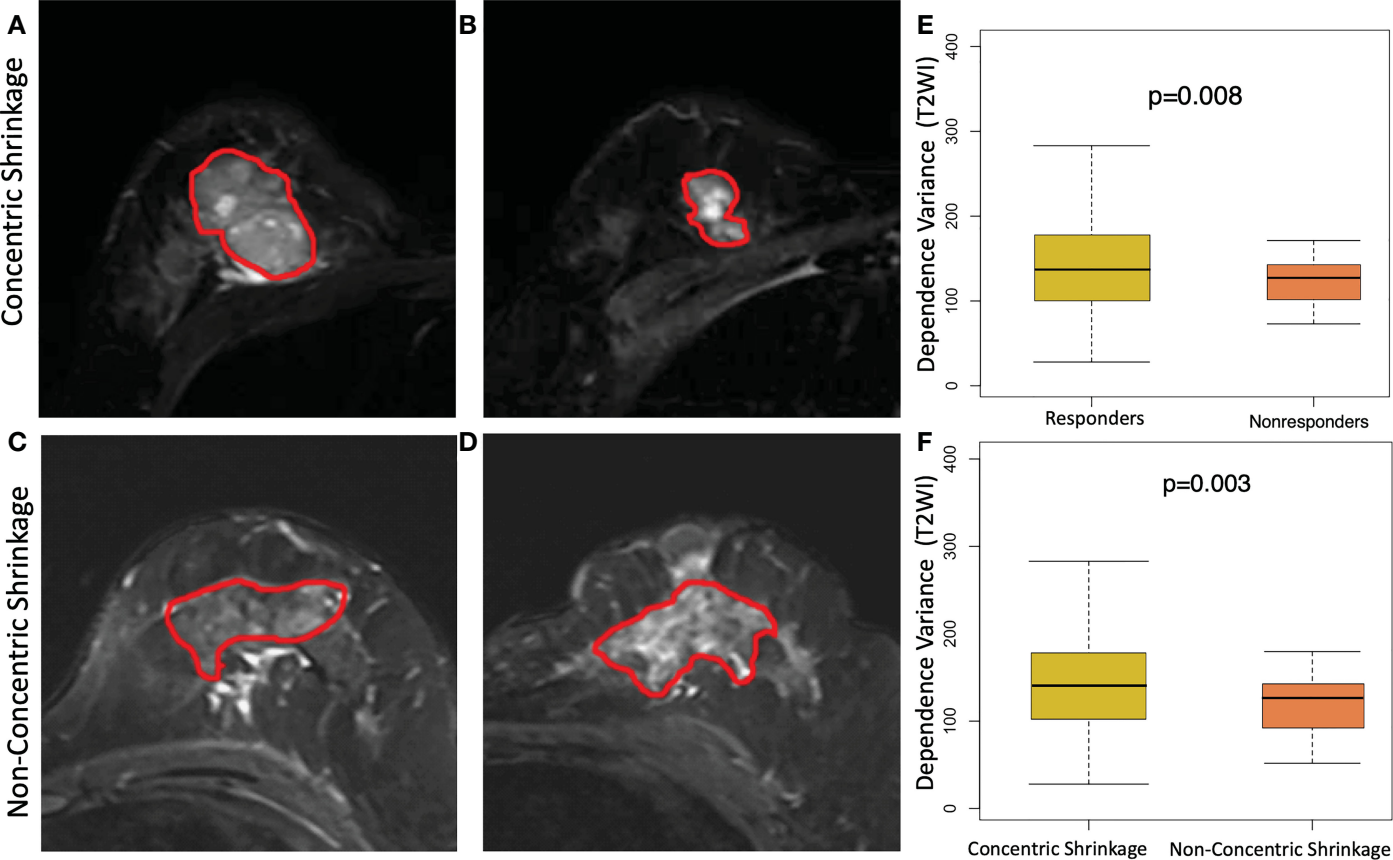
Texture feature of dependence variance feature at T2 weighting imaging (T2WI) for discriminating concentric shrinkage (CS) pattern/response and non-CS shrinkage pattern/nonresponse tumors. A 40-year-old woman with cancer in the right breast at **(A)** pretreatment and **(B)** posttreatment T2WI who has a good response to chemotherapy with a CS pattern. A 48-year-old woman with cancer in the left breast at **(C)** pretreatment and **(D)** posttreatment T2WI who had a poor response to chemotherapy with a non-CS pattern. The boxplot shows a significantly higher dependence variance feature value in the **(E)** clinical responder group than in the non-clinical responder group, and the same higher value was observed **(F)** in patients who were CS than in non-CS.

**Figure 3** shows an example of a texture feature (dependent variance) calculated from the subtraction image between the fifth and second postcontrast images (S52). A high level of this feature was significantly (p=0.002) correlated with the CS pattern. Moreover, a similar pattern of higher values in the clinical responders than in the non-clinical responders (p=0.004) was observed for this feature. In other words, a higher dependent variance value in terms of tumor heterogeneity is associated with a good response to NACT or CS. The T2WI-based dependence variance feature was also significantly higher in responders than in non-clinical responders (p=0.008) or higher in the CS pattern and non-CS pattern (p=0.003) (**Figure 4**).

**Figure 5:**
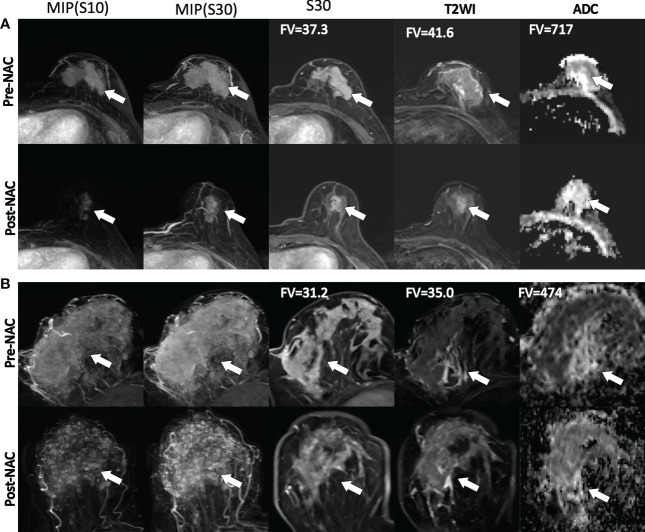
Illustration of the imaging features at pretreatment MRI and post NAC images. From the left to the right columns, the maximum intensity projection (MIP) at the first and third precontrast image series of the DCE-MRI, the third postcontrast image, the T2WI and ADC images are shown. **(A)** An example of a 47-year-old woman who had a good response to NACT and a concentric shrinkage (CS) pattern at preoperative and postoperative MRI examination. **(B)** An example of a 42-year-old woman who had a poor response or nonconcentric shrinkage pattern during NACT. The gray level dependence matrix (GLDM)-based dependence variance values from DCE and T2WI were 37.3 and 41.6, respectively, for the response and CS patients and 31.2 and 35.0, respectively, for the nonresponse and non-CS patients. The ADC values of the 10th percentile were 717 and 474 for the response/CS and the nonresponse/non-CS patients, respectively. FV, feature value.

Examples of the distributions of multiparametric image features in shrinkage patterns and treatment responses before treatment and after NACT are illustrated (**Figure 5**). **Figure 5A** shows an example of a patient with PR to NACT and a CS pattern, while **Figure 5B** illustrates a patient with nonresponse to treatment and diffuse decrease during NACT. A higher level of the difference variance value was observed in the clinical response/CS patient (37.3 and 41.6 for DCE-MRI and ADC images, respectively) than in the diffuse decrease/non-CS patient (31.2 and 35 for DCE-MRI and ADC images, respectively). Additionally, the high value of the 10th percentile tumor ADC in this patient was associated with a good response/CS pattern to NACT.

### Single parametric image-based predictive model

Radiomics from single parametric images was combined to establish predictive models. DCE-MRI series along with their subtraction maps and the T2WI and ADC images were assessed in the validation set separately (**Table 2**). Regarding DCE-MRI, the image subtraction-based predictive model had overall better prediction performance than that based on the original image series. Among these, the predictive model using the S50 image had the highest predictive performance for separating patients with pCR from those with non-pCR. The predictive model using the S52 image had the highest prediction performance for tumor clinical response (AUC=0.811, sensitivity of 0.654 at specificity of 1.0), and for tumor shrinkage prediction (AUC = 0.805, sensitivity of 0.820 at specificity of 0.750).

**Table 2:** DCE-MRI series-based model prediction performance evaluation.

	Pathological response			Clinical response			Shrinkage pattern		
Images	AUC	Sensitivity	Specificity	AUC	Sensitivity	Specificity	AUC	Sensitivity	Specificity
S0	0.661	0.5	0.860	0.724	0.719	0.750	0.675	0.440	1.000
S1	0.766	0.875	0.684	0.715	0.842	0.625	0.740	0.420	1.000
S2	0.763	0.625	0.930	0.724	0.719	0.750	0.618	0.420	0.875
S3	0.787	0.750	0.842	0.735	0.596	0.875	0.713	0.620	0.875
S5	0.797	0.625	0.895	0.743	0.439	1.000	0.703	0.680	0.750
S10	0.792	0.625	0.947	0.772	0625	0.912	0.748	0.780	0.750
S20	0.743	0.750	0.877	0.783	0.807	0.750	0.725	0.740	0.750
S30	0.718	0.50	0.947	0.739	0.860	0.625	0.777	0.500	1.000
S50	**0.821**	**0.750**	**0.860**	0.783	0.632	0.875	0.657	0.820	0.500
**S52**	0.757	0.625	0.930	**0.811**	**0.654**	**1.000**	**0.805**	**0.820**	**0.750**

CI, confidence interval; S0, precontrast; S1, S2, S3, S5 represent the first, second, third and fifth postcontrast images in DCE-MRI. S10, S20, S30 and S50 represent the subtraction map between the first, second, third and fifth postcontrast images and S0. S52 represents subtraction between the fifth and second postcontrast images. The rows with the highest AUC values are in bold.

The T2WI-based model achieved an AUC of 0.741 for predicting tumor shrinkage pattern and AUCs of 0.732 and 0.752 for the prediction of pathological and clinical responses to NACT, respectively (**Table 3**). The predictive model using radiomics from ADC had a relatively lower prediction performance, with AUCs of 0.625, 0.719 and 0.715 for the prediction of tumor pathologic response, clinical response and shrinkage pattern to NACT, respectively (**Table 3**).

**Table 3:** Predictive model performance using radiomics from single- and multiparametric images.

Images	AUC	95% CI	Sensitivity	Specificity	P
Tumor pathological response prediction					
T2WI	0.732	0.608-0.835	0.625	0.807	0.009
ADC	0.625	0.496-0.742	0.625	0.737	0.005
DCE-MRI	0.919	0.824-0.972	0.750	0.982	0.535
Multiparametric	0.943	0.856-0.985	0.875	0.912	–
Multiparametric +Luminal A	0.954	0.871-0.990	0.875	0.912	–
Tumor clinical response prediction					
T2WI	0.741	0.571-0.897	0.483	1.000	0.039
ADC	0.719	0.458-0.926	0.586	0.857	0.127
DCE-MRI	0.830	0.604-0.978	0.862	0.857	0.387
Multiparametric	0.919	0.845-0.978	0.845	1.000	–
Multiparametric +Luminal A	0.948	0.864-0.956	0.931	0.857	–
Tumor shrinkage pattern prediction					
T2WI	0.752	0.562-0.902	0.460	1.000	0.072
ADC	0.715	0.547-0.855	0.660	0.750	0.087
DCE-MRI	0.825	0.688-0.935	0.700	0.875	0.195
Multiparametric	0.905	0.792-0.985	0.800	0.875	–
Multiparametric + Luminal A	0.912	0.802-0.985	0.750	0.940	–

CI, confidence interval.

### Multiparametric image feature fusion-based prediction

**Figure 6:**
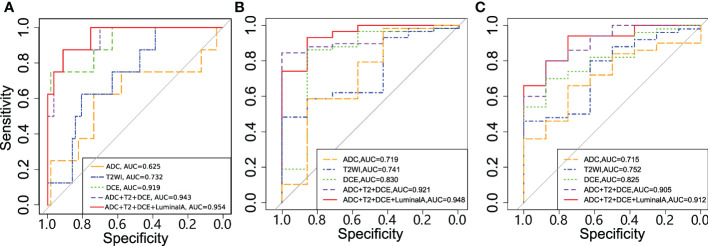
Receiver operating characteristic (ROC) curves for the models in predicting **(A)** tumor pathologic **(B)** clinical response and **(C)** shrinkage pattern.

Radiomics from the DCE-MRI series and multiparametric images were used and feature selection was performed. The models were evaluated in the validation set, which generated AUCs of 0.919, 0.830 and 0.825 for tumor pathologic response, clinical response and tumor shrinkage patterns, respectively (**Table 3**). When the features from the multiparametric images were combined with feature selection, the model generated a better performance in terms of AUCs of 0.943, 0.919 and 0.905 for these three tasks. The multiparametric image-based predictive model was significantly better than that based on T2WI in the prediction of tumor pathological and clinical response to NACT (p=0.009 and p=0.039, respectively). Thereafter, molecular subtype information was included as an additional feature along with features from ADC, T2WI and DCE. For tumor pathologic response prediction, the addition of luminal A, luminal B, HER2 and basal-like tumors generated AUCs of 0.954, 0.934, 0.952 and 0.950, while for tumor clinical response prediction, the AUCs were 0.948, 0.926, 0.913 and 0.914, respectively. For the tumor shrinkage pattern, the performances for the three tasks were improved while separately adding the four molecular subtypes with AUCs of 0.912, 0.905, 0.893 and 0.900. Among the four molecular subtypes, the inclusion of the luminal A subtype showed an optimal increment in the predictive model performance (**Figure 6**).

Feature importance in the multivariate predictive models for the two tasks was obtained (**Table 4**). Among them, 10 features were common in the multivariate predictive models, including the informational measure of correlation 1, cluster shade, run length nonuniformity normalized, short-run emphasis from S52, dependence variance and large dependence emphasis from T2WI, the dependence variance from S30, the 90th percentile from S10, and the maximum 2D diameter row and autocorrelation from the ADC. In the multivariate predictive model, the greatest number of features (5 out of 16) was obtained from the subtraction image map (i.e., S52) in DCE-MRI.

**Table 4:** Feature importance evaluation in tumor response and shrinkage pattern prediction.

Response to chemotherapy		Shrinkage pattern	
Feature	AUC	Feature	AUC
**IMC1^1^**	0.658	**Cluster shade^1^**	0.666
Cluster Shade^4^	0.611	Inverse difference normalized^5^	0.657
**Dependence variance**^4^	0.732	Run variance	0.665
**Dependence variance^3^**	0.723	**Run length nonuniformity normalized^1^**	0.714
**Cluster shade^1^**	0.624	**IMC1^1^**	0.656
Entropy^5^	0.712	**Autocorrelation**^5^	0.657
**90th Percentile^2^**	0.761	Dependence nonuniformity normalized^4^	0.661
Skewness^4^	0.732	Median^5^	0.753
Run variance^4^	0.652	**Dependence variance^3^**	0.652
Root mean squared**^2^**	0.694	Large area high gray level emphasis^4^	0.655
**Run Length nonuniformity normalized^1^**	0.692	**Maximum 2D diameter row**^5^	0.664
**Maximum 2D diameter row**^5^	0.712	**Short run emphasis^1^**	0.764
**Large dependence emphasis**^4^	0.588	Run percentage**^1^**	0.721
**Autocorrelation**^5^	0.713	**Dependence variance**^4^	0.657
**Short run emphasis^1^**	0.712	**90Percentile^2^**	0.678
High gray level run emphasis**^1^**	0.663	**Large dependence emphasis**^4^	0.634
**Collective features**	0.919	**Collective features**	0.905

IMC1, Informational Measure of Correlation 1.

^1^Image subtraction between the fifth and second postcontrast images (S52); ^2^ Image subtraction between the second and precontrast images (S10); ^3^ Image subtraction between the third and precontrast images (S30); ^4^T2 weighted image; ^5^Apparent diffusion coefficient image. Features that are common in the prediction model for response to chemotherapy and shrinkage pattern are in bold.

The predictive model performance was also evaluated by using nested 5-fold CV (**Supplementary Table 5**). From this table, similar results to those based on the testing dataset were observed. For example, T2WI based predictive model showed lower prediction performance in pathologic response, clinical response and shrinkage pattern with AUCs of 0.758, 0.708 and 0.731, respectively. The multiparametric image-based predictive model showed improved prediction performance with AUCs of 0.908, 0.868 and 0.843 for these three tasks.

## Discussion

A preoperative prediction of the tumor shrinkage pattern and the response to NACT in breast cancer was conducted using multiparametric radiomics from DCE-MRI, T2WI and ADC. Higher tumor heterogeneity evaluated by radiomic features, including the dependent variance value within the tumor, was associated with a good tumor response or CS pattern. The DCE-MRI subtraction image series-based model showed more discriminative power than either T2WI or ADC. Better performance was achieved by combining the multiparametric image features than by using a single series-based model. The addition of luminal A subtype information into the predictive model yields the highest performance.

A previous study used multiparametric images for the prediction of response to NACT and survival outcomes with 38 breast cancer samples (18). Tumor morphology, such as lesion size and volume distribution, is the most discriminative feature. A related multiparametric MRI study identified the tumor sphericity and mean absolute deviation feature value from T2WI and ADC, respectively, for predicting the response to treatment and shrinkage pattern (30). In our study, various types of features, including statistical, morphological and texture features, were identified from the multiparametric images. Among them, the dependence variance value was identified as the most discriminative feature. This feature measures the variance in gray level dependence size, which is defined as the number of connected voxels within a given distance that are dependent on the center voxel in the image. A high value of this feature reflects the high spread of the dependences of the tumor pixel value, which is correlated with a good response to the tumor or CS pattern during NACT. In a previous study, the addition of ER and node status into the radiomic model enhanced the prediction results for pCR or tumor shrinkage size (31). Our results demonstrated that the inclusion of the luminal A subtype data enhanced the predictive power of the model, which indicates a complementary information to imaging features. Women with luminal A breast cancer are less likely to achieve pCR after NACT than those with other molecular subtypes (32).

Comprehensive analysis of multiparametric images in our study demonstrated that the DCE-MRI-based model had higher performance (in terms of AUC) in tumor shrinkage and response prediction than T2WI and ADC images. A related study using multiparametric image radiomics reported a relatively lower prediction performance for a pretreatment ADC-based model (33), which is similar to our study. Compared with a lower sensitivity, the T2WI and ADC images showed a higher specificity in either the shrinkage pattern or response prediction. The combination of ADC, T2WI and DCE-MRI radiomics enhanced the overall model performance.

The predictive effectiveness for DCE-MRI was evaluated in different image series and maps, including the precontrast and subtraction images from different time points. It is interesting to note that the subtraction image had better predictive performance than either the precontrast or the postcontrast images. Among these, the subtraction images (e.g., S50 and S52) have better prediction performance than the other image series. This may be explained by the fact that the subtraction operation enhanced imaging evaluation of tumor angiogenesis and tumor heterogeneity by analyzing dynamic patterns of enhancement (34). The results may suggest that the washout pattern (35) calculated from the subtraction image between the last postcontrast images and the intermediate image (usually showing the highest enhancement) or precontrast image can be better than that using the original images.

### Limitations

Our study has several limitations. First, the number of patients in different groups (e.g., CS and non-CS) was imbalanced, which could have induced biased outcomes. Additional studies with larger datasets should be conducted to refine the results. Second, the imaging data were acquired from a single hospital/cohort with unified imaging protocols. The predictive model is limited to these specific imaging protocols.

## Conclusions

In summary, radiomics features of multiparametric images were used to predict tumor response and shrinkage patterns during NACT. In addition to molecular subtypes, the high imaging heterogeneity defined by radiomic features is associated with a good response or CS pattern. Further investigation in a larger and external validation dataset with different imaging protocols and longitudinal images (36) is warranted before our method can be used for breast cancer treatment management and decision making.

## Data availability statement

The original contributions presented in the study are included in the article/**Supplementary Material**. Further inquiries can be directed to the corresponding authors.

## Ethics statement

Ethical review and approval was not required for the study on human participants in accordance with the local legislation and institutional requirements. Written informed consent for participation was not required for this study in accordance with the national legislation and the institutional requirements.

## Author contributions

MF, TZ and LL designed the study. XW, JY, YL, KW, TX performed the image processing, machine learning methods, and statistical analyses. MF and LL wrote the manuscript. TZ and SC analyzed the data. All authors reviewed the manuscript.
