# Supplementary material for: Multiparametric MRI radiomics fusion for predicting the response and shrinkage pattern to neoadjuvant chemotherapy in breast cancer
Source: Front Oncol. 2023 May 3;13:1057841. doi: 10.3389/fonc.2023.1057841 (PMC10189126; doi:10.3389/fonc.2023.1057841)
Supplement: Supplementary file 1 [file DataSheet_1.docx]

Supplementary Table 1 Multiparameter Image Scan Parameters

| Image Parameter | DCE | T2WI | DWI | ADC |
| --- | --- | --- | --- | --- |
| Repetition time (TR) [ms] | 4.51 | 5800.0 | 7000.0 | 7000.0 |
| Echo time (TE) [ms] | 1.61 | 83.0 | 85.0 | 58.0 |
| Slice thickness (mm) | 1.2 | 6.0 | 6.0 | 6.0 |
| Flip angle (FA) [°] | 10 | 150 | 90 | 90 |
| Field of view (FOV) | 340×340 | 320×320 | 105×320 | 105×320 |
| Matrix | 896×896 | 640×640 | 72×220 | 72×220 |
| Pixel spacing (mm) | 0.379×0379 | 0.5×0.5 | 1.455×1.455 | 1.455×1.455 |

Supplementary Table 2. Radiomic feature illustration

| Shape features (n=14) | Elongation, Flatness, Mesh volume, Minor axis length, Sphericity, Surface area, Surface volume ratio, Voxel volume, Least axis length, Major axis length, Maximum 2D diameter column, Maximum 2D diameter row, Maximum 2D diameter slice, Maximum 3D Diameter |
| --- | --- |
| First order features (n=18) | 10Percentile, 90Percentile, Energy, Entropy, Interquartile range, Kurtosis, Maximum, Mean absolute deviation, Mean, Median, Minimum, Range, Robust mean absolute deviation, Root mean squared, Skewness, Total energy, Uniformity, Variance |
| Texture features (n=70) |  |
| Gray Level Co-occurrence Matrix (GLCM) features (n=24) | Autocorrelation, Cluster prominence, Cluster shade, Cluster tendency, Contrast, Correlation, Difference average, Difference entropy, Difference variance, Id, Idm, Idmn, IDN, IMC1, IMC2, Inverse variance, Joint average, Joint energy, Joint entropy, MCC, Maximum probability, Sum average, Sum entropy, Sum squares |
| Gray Level Run Length Matrix (GLRLM) Features (n=16) | Gray level nonuniformity, Gray level nonUniformity normalized, Gray level variance, High gray level run emphasis, Long run emphasis, Long run high gray level emphasis, Long run low gray level emphasis, Low gray level run emphasis, Run entropy, Run length nonuniformity, Run length nonUniformity normalized, Run percentage, Run variance, Short run emphasis, Short run high gray level emphasis, Short run low gray level emphasis |
| Gray Level Size Zone Matrix (GLSZM) Features (n=16) | Gray level nonuniformity, Gray level nonUniformity normalized, Gray level variance, High gray level zone emphasis, Large area emphasis, Large area high gray level emphasis, Large area low gray level emphasis, Low gray level zone emphasis, Size zone nonuniformity, Size zone nonUniformity normalized, Small area emphasis, Small area high gray level emphasis, Small area low gray level emphasis, Zone entropy, Zone percentage, Zone variance |
| Gray Level Dependence Matrix (GLDM) Features (n=14) | Dependence entropy, Dependence nonuniformity, Dependence nonUniformity normalized, Dependence variance, Gray Level nonuniformity, Gray level variance, High gray level emphasis, Large dependence emphasis, Large dependence high gray level emphasis, Large dependence low gray level emphasis, Low gray level emphasis, Small dependence emphasis, Small dependence high gray level emphasis, Small dependence low gray level emphasis |

**Supplementary Table 3**. Patient characteristics in the tumor response prediction dataset.

| Characteristics | All (n=216) | Development Set (n=151) | |  | | Validation Set (n=65) | |  | |
| --- | --- | --- | --- | --- | --- | --- | --- | --- | --- |
|  |  | Responder  (n=133) | Nonresponder  (n=18) | | P Value^*^ | Responder  (n=57) | Nonresponder  (n=8) | | P Value^*^ |
| Age(y) |  |  |  | | 0.320^a^ |  |  | | 0.239^a^ |
| Range | 26-67 | 27-66 | 33-65 | |  | 26-67 | 35-57 | |  |
| Median | 49 | 49 | 52 | |  | 51 | 48 | |  |
| Mean±std | 49.3±8.7 | 48.7±8.2 | 50.9±8.7 | |  | 50.3±10.0 | 47.1±6.3 | |  |
| Family history |  |  |  | | 1.000^c^ |  |  | | 0.675^c^ |
| No | 167(77%) | 102 | 14 | |  | 44 | 7 | |  |
| Yes | 49(23%) | 31 | 4 | |  | 13 | 1 | |  |
| Menopausal status | |  |  | | 0.890^b^ |  |  | | 0.672^b^ |
| Pre | 114(53%) | 73 | 9 | |  | 27 | 5 | |  |
| Post | 102(47%) | 60 | 9 | |  | 30 | 3 | |  |
| Maximum tumor diameter (cm) | | |  | | 0.643^a^ |  |  | | 0.769^a^ |
| Range | 0.5-14.5 | 0.5-9.0 | 2.5-7.8 | |  | 1.1-14.5 | 2.3-9.4 | |  |
| Median | 3.7 | 3.7 | 3.6 | |  | 3.8 | 3.2 | |  |
| Mean±std | 4.1±1.8 | 4.0±1.6 | 4.1±1.5 | |  | 4.4±2.3 | 4.1±2.4 | |  |
| Progesterone receptor | |  |  | | 0.178^b^ |  |  | | 0.456^c^ |
| Positive | 111(51%) | 71 | 6 | |  | 33 | 3 | |  |
| Negative | 105(49%) | 62 | 12 | |  | 26 | 5 | |  |
| Estrogen receptor | |  |  | | 0.142^b^ |  |  | | 0.709^c^ |
| Positive | 133(62%) | 87 | 8 | |  | 34 | 4 | |  |
| Negative | 83(38%) | 46 | 10 | |  | 23 | 4 | |  |
| Human epidermal growth factor receptor 2 | | |  | | 0.747^c^ |  |  | | 0.773^c^ |
| Positive | 83(38%) | 60 | 7 | |  | 15 | 1 | |  |
| Negative | 117(55%) | 62 | 9 | |  | 39 | 7 | |  |
| Unknown | 16(7%) | 11 | 2 | |  | 3 | 0 | |  |
| Ki-67 |  |  |  | | 0.695^c^ |  |  | | 0.373^c^ |
| High | 184(85%) | 117 | 17 | |  | 45 | 5 | |  |
| Low | 32(15%) | 16 | 1 | |  | 12 | 3 | |  |
| Molecular subtypes | |  |  | | 0.149^c^ |  |  | | 0.401^c^ |
| Luminal A | 20(9%) | 9 | 0 | |  | 10 | 1 | |  |
| Luminal B | 119(55%) | 84 | 8 | |  | 24 | 3 | |  |
| Basal-like | 41(19%) | 20 | 4 | |  | 13 | 4 | |  |
| HER2 | 36(17%) | 20 | 6 | |  | 10 | 0 | |  |
| Lymph node | |  |  | | 1.000^c^ |  |  | | 0.421^c^ |
| Positive | 168(78%) | 105 | 15 | |  | 43 | 5 | |  |
| Negative | 48(22%) | 28 | 3 | |  | 14 | 3 | |  |

^*^P value for responder versus nonresponder comparison. ^a^The data were tested using a t test; ^b^The data were tested using the chi-squared test. ^c^The data were tested using Fisher’s exact test.

Supplementary table 4. Patient characteristics for the tumor shrinkage pattern

| Characteristics | All (n=193) | Development Set (n=135)  (n=135) | | | Validation Set (n=58)  (n=58) | | |
| --- | --- | --- | --- | --- | --- | --- | --- |
|  |  | CS  (n=116) | Non-CS  (n=19) | P^*^ | CS  (n=50) | Non-CS  (n=8) | P^*^ |
| Age(y) |  |  |  | 0.890^a^ |  |  | 0.637^a^ |
| Range | 26-67 | 26-66 | 33-65 |  | 29-67 | 40-57 |  |
| Median | 50 | 50 | 48 |  | 50 | 51 |  |
| Mean±std | 49.5±8.7 | 49.2±8.3 | 49.3±8.8 |  | 49.6±8.4 | 50.6±5.3 |  |
| Family history | |  |  | 0.381^b^ |  |  | 0.616^c^ |
| No | 147(76%) | 83 | 16 |  | 42 | 6 |  |
| Yes | 46(24%) | 33 | 3 |  | 8 | 2 |  |
| Menopausal status | |  |  | 0.696^b^ |  |  | 0.464^c^ |
| Pre | 99(51%) | 58 | 11 |  | 27 | 3 |  |
| Post | 94(49%) | 58 | 8 |  | 23 | 5 |  |
| Maximum tumor diameter(cm) | | |  | 0.471^a^ |  |  | 0.256^a^ |
| Range | 0.5-14.5 | 0.5-14.5 | 2.3-9.4 |  | 2.0-7.4 | 2.4-5.6 |  |
| Median | 3.7 | 3.7 | 4.0 |  | 3.7 | 3.4 |  |
| Mean±std | 4.1±1.8 | 4.2±2.0 | 4.5±1.9 |  | 4.0±1.4 | 3.4±1.0 |  |
| PR |  |  |  | 0.147^b^ |  |  | 0.719^c^ |
| Positive | 93(48%) | 61 | 6 |  | 23 | 3 |  |
| Negative | 100(52%) | 55 | 13 |  | 27 | 5 |  |
| ER |  |  |  | 0.188^b^ |  |  | 1.000^c^ |
| Positive | 116(60%) | 71 | 8 |  | 32 | 5 |  |
| Negative | 77(40%) | 45 | 11 |  | 18 | 3 |  |
| HER2 |  |  |  | 0.407^c^ |  |  | 1.000^c^ |
| Positive | 73(38%) | 45 | 5 |  | 20 | 3 |  |
| Negative | 106(55%) | 61 | 11 |  | 29 | 5 |  |
| Unknown | 14(7%) | 10 | 3 |  | 1 | 0 |  |
| Ki-67 |  |  |  | 1.000^c^ |  |  | 0.099^c^ |
| High | 168(87%) | 102 | 17 |  | 44 | 5 |  |
| Low | 25(13%) | 14 | 2 |  | 6 | 3 |  |
| Lymph node | |  |  | 0.406^c^ |  |  | 0.342^c^ |
| Positive | 148(77%) | 86 | 16 |  | 41 | 5 |  |
| Negative | 45(23%) | 30 | 3 |  | 9 | 3 |  |

*P value for CS versus non-CS comparison; ^a^The data were tested using a t test; ^b^The data were tested using the chi-squared test.

^c^The data were tested using Fisher’s exact test. ER=estrogen receptor; PR=progesterone receptor; HER2=human epidermal growth factor receptor 2; Ki-67=Antigen identified by monoclonal antibody Ki-67.

| Images | Clinical response | Pathological response | Shrinkage pattern |
| --- | --- | --- | --- |
| T2WI | 0.758 | 0.708 | 0.731 |
| ADC | 0.761 | 0.644 | 0.735 |
| DCE-MRI | 0.860 | 0.844 | 0.794 |
| Multiparametric | 0.908 | 0.868 | 0.843 |
| Multiparametric +Luminal A | 0.913 | 0.876 | 0.854 |

Supplementary table 5. Predictive model performance using radiomics from single- and multiparametric images under nested 5-fold cross validation.


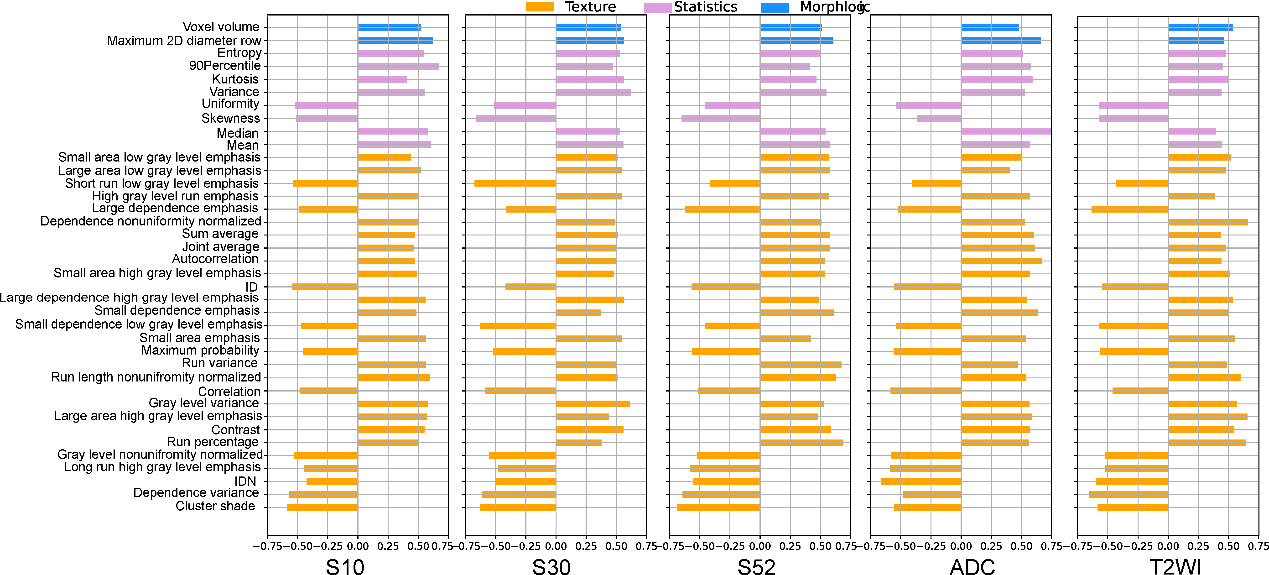


**Supplementary Figure 1**. Individual feature performance for predicting shrinkage pattern to treatment. The bar plot is drawn on the right side if a higher value was observed in concentric shrinkage (CS) than in non-CS, while the bar plot is drawn on the right side if the opposite was true. S10, S20, S30 and S50 represent the subtraction map between the first, second, third and fifth postcontrast images and precontrast image. S52 represents subtraction between the fifth and second postcontrast images.
